# Automated mental health screening in pediatric lupus: associations with disease features and treatment

**DOI:** 10.3389/fped.2024.1427543

**Published:** 2024-10-08

**Authors:** Lauren Harper, Stacy P. Ardoin, Alana Leever, Kyla Driest, Vidya Sivaraman, Alysha J. Taxter

**Affiliations:** ^1^ Department of Pediatric Rheumatology, Nationwide Children’s Hospital, Columbus, OH, United States; ^2^ Department of Pediatrics, The Ohio State University School of Medicine, Columbus, OH, United States; ^3^ Department of Clinical Informatics, Nationwide Children’s Hospital, Columbus, OH, United States

**Keywords:** lupus, pediatric, depression, mental health, screening, patient health questionnaire, patient-reported outcomes, informatics

## Abstract

**Introduction:**

Patients with childhood-onset systemic lupus erythematosus (c-SLE) have higher rates of depression than their peers, which has been associated with worse medical outcomes. Therefore, it is imperative that their mental health be addressed. We utilized quality improvement (QI) methodology to automate mental health screening for patients with lupus within a pediatric rheumatology clinic. The retrospective cohort study aims to evaluate the association between mental health screening outcomes and demographics, medications, and disease activity measures in patients with childhood lupus.

**Methods:**

The mental health QI team at a quaternary pediatric rheumatology center implemented an automated process for mental health screening in patients with c-SLE. Patients seen between 2017 and June 2023 with a diagnosis of c-SLE were identified using International Classification of Disease -Clinical Modification (ICD-CM) codes. Disease activity was assessed with the Systemic Lupus Erythematosus Disease Activity Index 2000 (SLEDAI 2K). Medications were identified on outpatient and inpatient orders for conventional synthetic and biologic disease-modifying anti-rheumatic drugs, hydroxychloroquine, corticosteroids, and aspirin. Mental health screening was accomplished with the Patient Health Questionnaire (PHQ). Descriptive statistics, univariate and multivariate linear regression were used.

**Results:**

Between January 2017 and June 2023, 117 patients with c-SLE (41% with lupus nephritis) completed 534 total screenings. Each patient completed PHQ screenings, a median of 5 [interquartile range 2, 6] times. Screening increased when the screening process was automated. Those who were Black, female, or prescribed leflunomide, mycophenolate, and corticosteroids had higher PHQ scores.

**Conclusions:**

Mental health support is essential for patients with chronic rheumatologic diseases such as SLE. Sustainable processes for quickly identifying depression are needed for optimal care of patients with SLE. Our process of automated, streamlined mental health screening successfully increased the screening of patients with SLE at every visit and led to timely interventions for positive PHQ scores. Higher PHQ scores were correlated with patients on leflunomide, mycophenolate, and corticosteroids. Future research should identify modifiable risk factors for high PHQ scores that the medical team can target.

## 1 Introduction

Addressing mental health is essential to optimally care for and treat patients with systemic lupus erythematosus (SLE). Pediatric and adult patients with SLE have higher rates of depression and anxiety than the general population (1–5). Children with SLE have 2.9 times increased odds of being diagnosed with depression and have 5.4 times increase in suicidal ideation (4). The reported prevalence of depression in childhood-onset SLE (c-SLE) is 20%–59% (5), compared to 24% in adults (6).

In addition to its prevalence, pediatric patients with more severe depression have increased lupus disease activity, cardiovascular disease, physical disability, suicidal ideation, premature mortality, and lower educational attainment (7). In general, patients with depression are three times more likely to have medication non-compliance than their non-depressed counterparts (8), and increased medication non-adherence is associated with worsening depression symptoms in patients with c-SLE (5).

Given these outcomes, a survey of members of the Childhood Arthritis and Rheumatology Research Alliance (CARRA) reported that 95% of responding pediatric rheumatologists supported mental health screening every 6–12 months. However, only 7% of this cohort routinely screened symptomatic patients, and only 2% screened all patients with a standardized, validated tool (9). While, in general, providers in pediatric rheumatology recognize the need for addressing mental health, implementation of screening is lacking. This discrepancy is likely due, in part, to inefficient screening methods and the fact that the current screening approaches may not be sufficient to address mental health needs in this population (10).

In this study, we describe the quality improvement efforts at a large quaternary children's hospital to automate and streamline mental health screening, making this essential and potentially time-consuming process feasible in a busy clinic setting. We also compared these mental health screening scores to patient demographics, immunosuppressive medications, and lupus disease activity.

## 2 Materials and methods

### 2.1 Setting

Nationwide Children's Hospital (NCH) is a large pediatric quaternary care academic medical center. The rheumatology team at NCH comprises pediatric rheumatologists, pediatric rheumatology fellows, a nurse practitioner, a social worker, nurses, a pharmacist, and a clinical psychologist. Our team utilized quality improvement methodology to develop an automated screening process to assess depression in patients with c-SLE (11, 12). We then retrospectively evaluated the data collected between January 2017 and June 2023. This study was approved by the Institutional Review Board (STUDY00003317).

Patient Population: Patients evaluated at a large quaternary care hospital outpatient rheumatology clinic with a diagnosis of c-SLE were identified using the respective International Classification of Disease—Clinical Modification (ICD-CM) codes 710 (ICD9) and M32 (ICD10).

### 2.2 Patient characteristics

Patient demographics were extracted from the electronic health record, which included sex, race, and ethnicity. Race was categorized as White, Asian, Black, Multiple, or Other/Unknown. Nephritis was defined as a patient with an ICD-9 or ICD-10 code for lupus nephritis. The specific ICD codes utilized were 710.0, 583.81, 710.0, 583.89, M32.14, and M32.15. Date of diagnosis and date of Patient Health Questionnaire (PHQ)-8 completion were recorded, and disease duration was calculated.

### 2.3 Disease activity

Systemic Lupus Erythematosus Disease Activity Index 2000 (SLEDAI 2 K) was calculated at each standard of care visit (13). In some instances, the SLEDAI 2 K was not fully completed when initially recorded due to pending lab values during clinical care and was later calculated via automated processes. For urinary values, if epithelial cells were present, the sample was considered contaminated, and hematuria and pyuria were not recorded unless this was documented as due to active disease by the provider. Similarly, if hematuria was present, manual chart review was completed to evaluate menstruation status. Patient and provider-reported disease activity assessments were recorded on a standardized 0–10 scale, with higher score indicating worse disease (14). These scores have been recommended by the American College of Rheumatology and the Outcome Measures in Rheumatology Consensus Initiative for use to more fully evaluate the overall health of patients with rheumatologic conditions (15, 16).

### 2.4 Medications

Outpatient and inpatient orders for conventional synthetic and biologic disease-modifying anti-rheumatic drugs, hydroxychloroquine, corticosteroids, and aspirin were identified; the first and last orders for each medication were identified, and consistent use was assumed. Cyclophosphamide exposure was defined as the first and last dates of consecutive infusions, with no more than 120 days between administered doses, plus 28 days from the last dose; this broad administration window would account for medication being held, such as in the case of an infection. Rituximab exposure was defined as the first and last dates of consecutive rituximab infusion occurring within 40 days of each administered dose plus six months from the last dose, as we assume rituximab would have an effect for approximately six months after the last dose. One patient could have multiple courses of cyclophosphamide and/or rituximab exposures.

### 2.5 Mental health screening with patient health questionnaire (PHQ)-8

Our team initially utilized the PHQ-9 for depression screening. The PHQ-9 includes a self-harm question; our questionnaire included additional self-harm questions including “Has there been a time in the past month when you had serious thoughts about ending your life?” and “Have you ever, in your whole life, tried to kill yourself or made a suicide attempt?”. In 2023, the PHQ-8 and Ask Suicide Questionnaire replaced the PHQ-9 and additional self-harm questions, to be more complete when evaluating suicidality. Of those who completed the PHQ-9, their scores were recalculated to only capture PHQ-8 questions. The PHQ-8 will hereafter be referred to as PHQ. Due to the critical need to act on high scores or an indication of suicidality, a PHQ was not given to the patient until he or she physically arrives at clinic. PHQ scores of 0–4, 5–9, 10–14, and 15–19, and 20 or greater indicate none, mild, moderate, moderately severe, and severe depression, respectively (17). Previous quality improvement interventions included increasing rheumatology providers’ awareness of screening by discussing it at staff meetings, streamlining the workflow of mental health screening for social work and the nursing team, integration of identifying patients to be screened into nursing pre-visit planning, and increasing patient and family awareness of the screening project and mental health issues in rheumatology. These interventions resulted in annual, routine PHQ screening in 2017 on paper for all English-speaking patients with lupus ≥12 years old; responses were transcribed into the electronic health record (11). In 2021, our final QI cycle automated the screening process. The PHQ questionnaire was transitioned from a paper form to being delivered electronically on a tablet to all English-speaking patients with lupus ≥12 years of age at every visit; this questionnaire was automatically assigned to the clinic encounter and only available upon checking into clinic.

**Figure 1:**
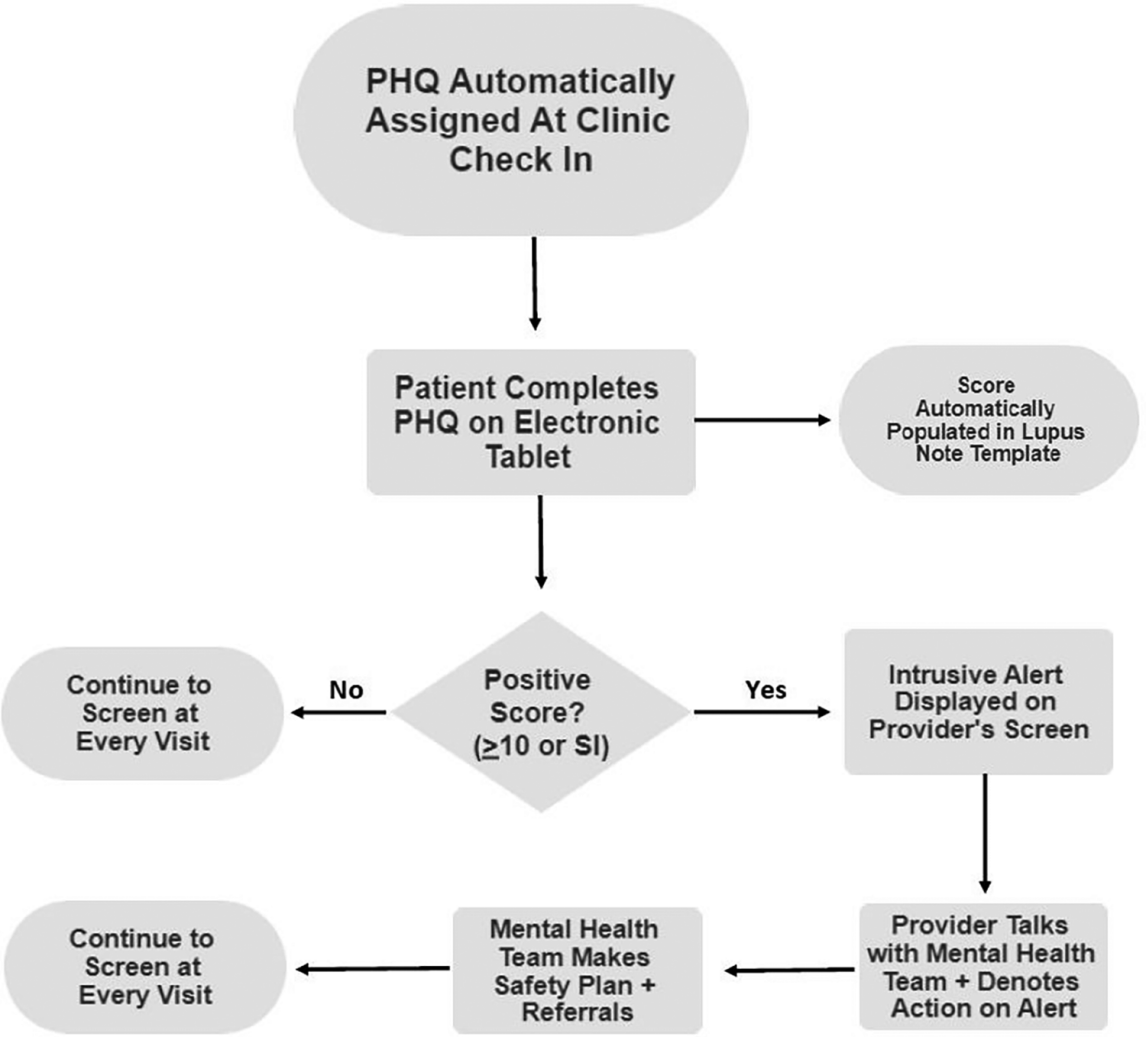
Clinical workflow for critical alerts. PHQ, Patient Health Questionnaire; SI, suicidal ideation.

After full integration into the electronic health record (EHR), PHQ scores were automatically calculated and populated in the clinic note with a drop-down menu of options indicating the action taken. Patients with a PHQ score of 5–9 were provided a handout focusing on psychoeducation and contact information for the rheumatology psychosocial team, including social worker and psychologist. A PHQ score of ten or higher would trigger an intrusive pop-up alert in the EHR when providers, including physicians, nurse practitioners, social workers, and psychologists, open the patient's chart. The alert would then be acknowledged, and the provider would address the concern or contact a social worker or psychologist if they were unaware. A social worker or rheumatology psychologist would then meet with the patient during the clinic visit. A thorough suicide risk assessment would be done with the Ask Suicide Questionnaire and Columbia Suicide Severity Rating Scale, and the patient would be offered a behavioral health referral (Figure 1) (18, 19).

### 2.6 Provider feedback

Five months after complete automation, providers were asked “How satisfied are you with the current automated depression screening for patients with lupus” on a 0–10 ordinal scale, with anchors of “0-Not Satisfied” and “10-Very Satisfied”.

### 2.7 Statistical analysis

Summary statistics describing the population are reported as median [interquartile range (IQR)] and count (percent). Univariate regression evaluated the association between PHQ scores, patient, disease characteristics, activity scores, and treatments. Variables with *p* < 0.20 in univariate were evaluated in multivariate linear regression modeling. Analysis was completed using Stata 16.0.

## 3 Results

**Figure 2:**
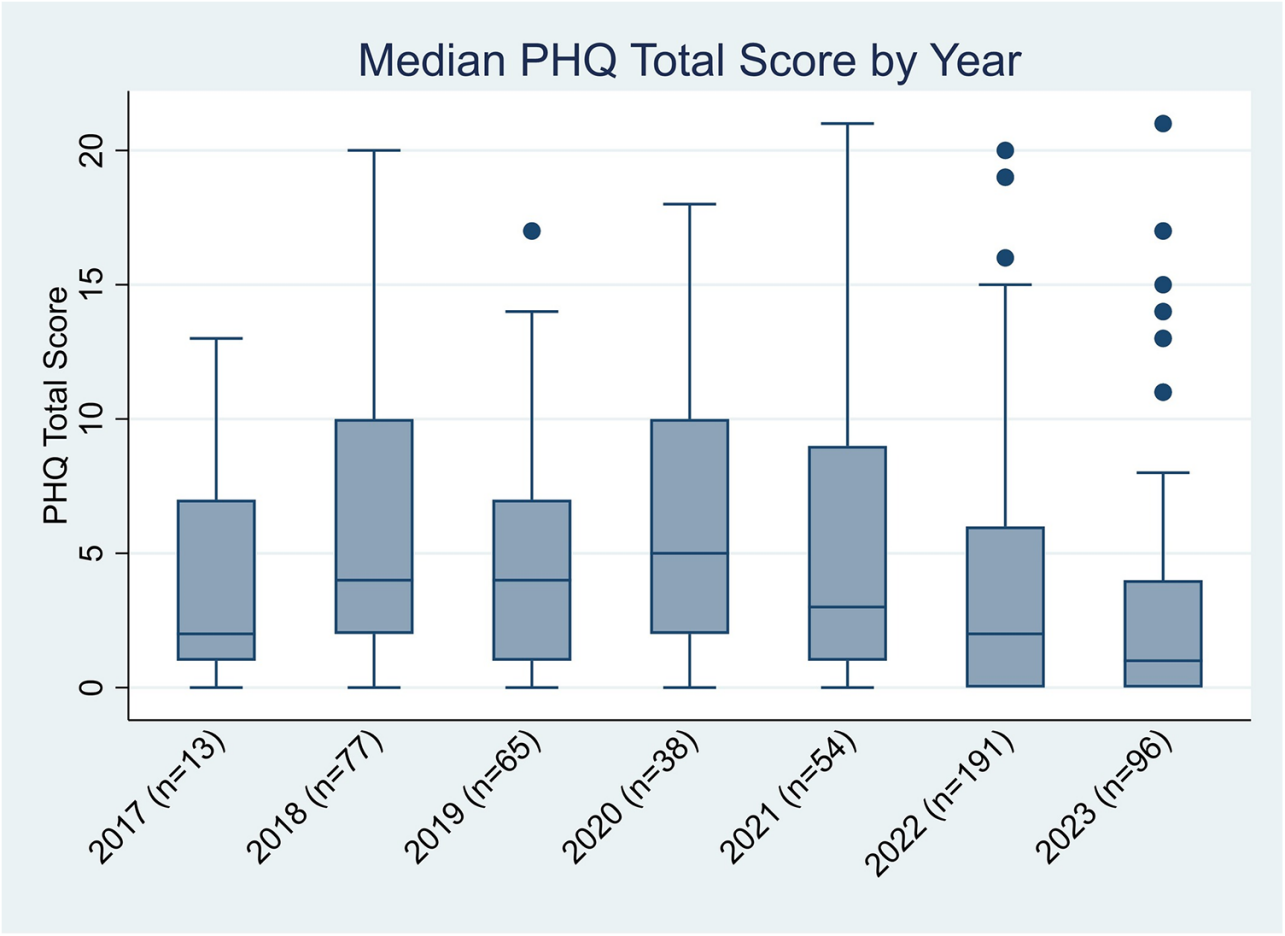
Median PHQ scores by year. PHQ, Patient Health Questionnaire; SLE, systemic lupus erythematosus.

One hundred seventeen unique patients (41% with lupus nephritis) completed 534 screenings (Table 1). Each patient completed PHQ screenings a median of 5 [IQR: 2, 6] times. Screening frequency increased after electronic implementation. A mean of 50 screens were completed annually between 2017 and 2021; screens increased to 191 in 2022 and the first two quarters of 2023, with a median score of 2 [0, 7]. Figure 2 depicts median PHQ scores by year. Those with a new diagnosis within the prior 6 months had an average PHQ score of 2 [0,6] whereas those diagnosed more than 6 months ago had an average PHQ score of 3 [0, 7]. There was no statistical significance between the two groups (*p* = 0.45). Of the patients who completed a PHQ, eight (4%) reported a suicide attempt, and two (1%) had suicidal thoughts within the past month. PHQ scores were 347 (64%), 99 (19%), 64 (12%), 20 (4%), and 4 (1%) indicating none, mild, moderate, moderately severe, and severe scores, respectively. After complete automation, intrusive alerts fired appropriately for PHQ scores 10 or higher. This alert fired 84 times at 23 visits. It occurred from 1 to 9 times per visit, as it would be triggered each time a provider newly entered the chart. A provider acted on the alert at every visit. During all but three visits, either a social worker or psychologist met with the patient to follow up on the elevated PHQ score. In these three visits without follow-up, the patients were already connected with psychology, psychiatry, or counseling, and none were actively suicidal. Of the 12 providers surveyed, the median satisfaction of the new automated screening was 10 [9, 10].

**Table 1:** Demographics, disease activity, and mental health screening by presence of nephritis.

	All patients (*N* = 534)	Without nephritis (*N* = 316)	With nephritis (*N* = 218)	*P*-value
PHQ Score by Year				0.21
2017	2 [1, 7]	4 [1, 7]	2 [1, 5]	
2018	4 [2, 10]	4 [1, 10]	4 [2, 10]	
2019	4 [1, 7]	2 [0, 7]	5 [3, 9]	
2020	5 [2, 10]	4 [1, 8]	7 [3, 10]	
2021	3 [1, 9]	3 [1, 10]	2 [0, 7]	
2022	2 [0, 6]	0 [0, 5]	3 [0, 7]	
2023	1 [1, 4]	0 [0, 4]	3 [0, 7]	
Age	17 [16, 19]	18 [16, 19]	17 [15, 19]	<0.01
Female sex	434 (81%)	257 (81%)	177 (81%)	0.98
Race				0.02
Asian	57 (11%)	33 (11%)	24 (11%)	
Black	193 (36%)	121 (38%)	72 (33%)	
Multiple	33 (6%)	18 (6%)	15 (7%)	
Other/Unknown	34 (6%)	12 (3%)	22 (10%)	
White	217 (41%)	132 (42%)	85 (39%)	
Hispanic ethnicity	49 (9%)	23 (7%)	26 (11%)	0.07
Patient global^a^	1 [0, 4]	1 [0, 4]	1 [0, 3]	0.53
Provider global^b^	1 [0, 2]	1 [0, 1]	1 [0, 3]	<0.01
SLEDAI 2K^c^	2 [0, 6]	2 [0, 4]	4 [0, 8]	<0.01

PHQ, patient health questionnaire; SLEDAI 2K, systemic lupus erythematosus disease activity score 2000.

^a^ Available in 145 encounters.

^b^ Available in 318 encounters.

^c^ Available in 374 encounters.

In univariate analysis, leflunomide, hydroxychloroquine, mycophenolate, corticosteroids, female sex, Asian and Black race, Hispanic ethnicity, patient global, provider global, and SLEDAI 2K were associated with higher PHQ scores (Table 2). In multivariate analysis, Black race and patient global scores were associated with higher PHQ scores provider (Table 3).

**Table 2:** Univariate linear regression of Patient Health Questionnaire 8 by medication use, demographics, and disease activity score.

	*β*-coefficient (95% CI)	*P*-value
Hydroxychloroquine	0.95 (0.08, 1.81)	0.03
Sulfasalazine	1.82 (−1.58, 5.24)	0.29
Leflunomide	13.83 (1.31, 23.34)	<0.01
Methotrexate	−1.00 (−2.47, 0.47)	0.18
Mycophenolate	1.92 (1.01, 2.82)	<0.01
Azathioprine	0.17 (−0.87, 1.21)	0.75
Tacrolimus	3.07 (−1.72, 7.87)	0.21
Intravenous immunoglobulin	2.81 (−3.97, 9.59)	0.42
Corticosteroids	1.31 (0.49, 2.14)	<0.01
Abatacept	1.85 (−0.84, 4.53)	0.18
Aspirin	−0.47 (−2.64, 1.72)	0.68
Belimumab	1.20 (−0.42, 2.84)	0.15
Rituximab	0.89 (−0.85, 2.63)	0.32
Cyclophosphamide	1.06 (−0.74, 2.85)	0.25
Age	0.04 (−0.12, 0.20)	0.60
Female sex	1.47 (0.42, 2.53)	<0.01
Race		
White	Reference	Reference
Asian	−1.70 (−3.06, −0.33)	0.02
Black	2.40 (1.49, 3.31)	<0.01
Multiple	0.19 (−1.53, 1.91)	0.83
Other/Unknown	−0.60 (−2.29, 1.10)	0.49
Hispanic ethnicity	1.54 (0.12, 2.97)	0.03
Patient global	0.77 (0.60, 1.15)	<0.01
Provider global	0.55 (0.21, 0.90)	<0.01
SLEDAI 2K	0.17 (0.07, 0.26)	<0.01

CI, confidence intervals; SLEDAI 2K, systemic lupus erythematosus disease activity score 2000.

**Table 3:** Multivariate linear regression of Patient Health Questionnaire by medication use, demographics, and disease activity score.^a^

	*β*-coefficient (95% CI)	*P*-value
Hydroxychloroquine	0.91 (−0.58, 2.39)	0.23
Leflunomide	—	—
Methotrexate	0.69 (−1.35, 2.75)	0.50
Mycophenolate	1.21 (−0.22, 2.65)	0.10
Corticosteroids	−0.22 (−1.66, 1.21)	0.76
Abatacept	−6.27 (−11.49, 1.04)	0.02
Belimumab	−1.19 (−3.74, 1.36)	0.35
Female sex	−0.34 (−1.86, 1.17)	0.66
Race		
White	Reference	Reference
Asian	1.63 (−0.22, 3.47)	0.08
Black	2.28 (0.77, 3.79)	<0.01
Multiple	1.82 (−1.87, 5.51)	0.33
Other/Unknown	4.52 (0.80, 8.23)	0.02
Hispanic ethnicity	1.59 (−1.77, 4.96)	0.35
Patient global	0.81 (0.52, 1.09)	<0.01
Provider global	0.52 (0.08, 0.96)	0.02
SLEDAI 2K	−0.07 (−0.20, 0.06)	0.20

CI, confidence intervals; SLEDAI 2K, systemic lupus erythematosus disease activity score 2000.

^a^ Model reduced to 132 encounters.

## 4 Discussion

Our study shows that depression is highly prevalent in those with c-SLE, as more than one-third of PHQ screenings were at least 10 or higher, which indicates a likelihood ratio of 7.1 and specificity of 88% for depression per a PHQ validity study (17). We sought to streamline the mental health screening process to better detect depression, thereby optimizing care for children with SLE. Automating PHQ screening and embedding the process in the electronic medical record ensured consistent screening and that every positive screen was addressed, reducing the burden on clinic staff. This process solves many of the frequently cited obstacles in the CARRA mental health survey: time constraints and increased burden on staff (9). Our embedded collaborative psychology and social work teams within the rheumatology clinic were instrumental to complete these screenings as they allowed us to act upon positive screens without the need for transfer to the emergency room or behavioral health to obtain additional evaluation and safety plan initiation. We successfully increased screening from an annual screen to screening at every visit with the implementation of automated questionnaires. One of the biggest challenges we faced was the COVID-19 pandemic and the need for telehealth visits and the screening frequency decreased.

This study also evaluated relationships between mental health screening results, demographics, medications, and lupus disease activity measures. Our study showed that leflunomide, mycophenolate, and corticosteroids, was associated with higher PHQ scores, i.e., worse mental health. Steroid use is associated with mood dysregulation and depression (20), and patients requiring steroids typically use this medication early in the disease course or during flares. Therefore, steroids are needed during more stressful times when patients’ disease is active, and they do not feel well. However, we did not see similar associations with cyclophosphamide or rituximab, commonly used during active disease. Interestingly, higher PHQ scores and belimumab use were not significantly correlated despite documented concerns that belimumab may worsen depression and suicidality (21). Our results support those of a meta-analysis of randomized control trials of belimumab use for patients with SLE, which did not find that depression or suicidality increased in patients taking belimumab (22). Another contributing factor may be that prescribers avoid starting belimumab for patients with known depression, who would be at higher risk for elevated PHQ scores.

We also found that lower patient global and provider global scores were associated with lower PHQ scores. This finding is not surprising, given that lower scores indicate less active disease, and when patients are less symptomatic and feel better, they may have lower rates of depression. This is in alignment with previous studies showing that those in remission have less depression, less anxiety, and improved health-related quality of life (23, 24).

Patients who were Black were significantly more likely to have high PHQ scores (*p* < 0.01). Although this relationship between depression and increased PHQ scores has been previously noted in minority populations, minorities are less likely to receive counseling or other support services for their mental health than non-Latino White children (25). This finding likely has multifactorial implications involving social determinants of health and inequities in the healthcare system. For example, another study found that being a person of color, attaining a lower level of education, being unmarried, not having medical insurance, and being unemployed were all associated with higher PHQ scores. Interestingly, when accounting for other socioeconomic factors, Black race was associated with higher PHQ scores, which highlights the complexity of evaluating social constructs (26). Given this intricate interplay, social determinants of health must be considered when addressing mental health. In addition, more efforts must address disparities in mental health care between White populations and people of color.

Few patients reported a suicide attempt and suicidal thoughts, 8 (4%) and 2 (1%), respectively. Consistent screening would identify these patients so interventions could be quickly implemented. While these results are serious and necessitate action, this small number also highlights that, based on our results, we would not expect an overwhelming number of critical screens in a pediatric rheumatology office.

This study was limited by its setting, i.e., a single-center initiative in a large children's hospital with a social worker and psychologist embedded in the rheumatology clinic. Similar screening can be performed without these resources using automated screening with provider alerts for critical results. However, timely access to mental health resources will be vital to implementing a similar process in other institutions. The PHQ assesses symptoms of depression and suicidality, but it does not diagnose major depressive disorder per the Diagnostic and Statistical Manual of Mental Disorders (27). However, the PHQ is commonly used to screen for mental health concerns and is a validated screening tool (17).

Future research should assess whether modifiable risk factors can be identified for patients with SLE and depression. Analysis of social determinants of health specific to mental health scores may be informative. This analysis would ideally allow the medical team to intervene on those risk factors before patients develop significant depression or suicidality. In summary, it is clear that mental health support is essential for patients with chronic rheumatologic diseases such as SLE. Sustainable processes for timely identification of depression are needed to best take care of patients with SLE. Our process of automated, streamlined mental health screening successfully led to an increase in screening patients with lupus at every visit and in providing timely interventions for positive PHQ scores. Higher PHQ scores were correlated with patients being on leflunomide, mycophenolate, and corticosteroids. Future research should seek to identify modifiable risk factors for high PHQ scores that can be targeted by the medical team and to develop streamlined pathways for intervention.

## 5 Conclusions

Mental health support is essential for patients with chronic rheumatologic diseases such as SLE. Sustainable processes for quickly identifying depression are needed to best take care of patients with SLE. Our process of automated, streamlined mental health screening successfully increased screenings of patients with lupus at every visit and provided timely interventions for positive PHQ scores. Higher PHQ scores were correlated with patients on leflunomide, mycophenolate, and corticosteroids. Future research should identify modifiable risk factors for high PHQ scores so the medical team can target and develop streamlined intervention pathways.

## Data Availability

The original contributions presented in the study are included in the article/Supplementary Material, further inquiries can be directed to the corresponding authors.
